# Nanopore-based kinetics analysis of individual antibody-channel and antibody-antigen interactions

**DOI:** 10.1186/1471-2105-8-S7-S20

**Published:** 2007-11-01

**Authors:** Stephen Winters-Hilt, Eric Morales, Iftekhar Amin, Alexander Stoyanov

**Affiliations:** 1 The Research Institute for Children, 200 Henry Clay Ave., New Orleans, LA 70118, USA; 2 Department of Computer Science, University of New Orleans, New Orleans, LA, 70148, USA

## Abstract

**Background:**

The UNO/RIC Nanopore Detector provides a new way to study the binding and conformational changes of individual antibodies. Many critical questions regarding antibody function are still unresolved, questions that can be approached in a new way with the nanopore detector.

**Results:**

We present evidence that different forms of channel blockade can be associated with the same antibody, we associate these different blockades with different orientations of "capture" of an antibody in the detector's nanometer-scale channel. We directly detect the presence of antibodies via reductions in channel current. Changes to blockade patterns upon addition of antigen suggest indirect detection of antibody/antigen binding. Similarly, DNA-hairpin anchored antibodies have been studied, where the DNA linkage is to the carboxy-terminus at the base of the antibody's Fc region, with significantly fewer types of (lengthy) capture blockades than was observed for free (un-bound) IgG antibody. The introduction of chaotropic agents and its effects on protein-protein interactions have also been observed.

**Conclusion:**

Nanopore-based approaches may eventually provide a direct analysis of the complex conformational "negotiations" that occur upon binding between proteins.

## Background

### The highly stable Alpha-Hemolysin protein channel

The alpha-Hemolysin toxin is produced by the bacteria *Staphylococcus aureus*. The alpha-Hemolysin channel is a heptamer, a seven member molecular complex. Each alpha-hemolysin monomer is water soluble and on the membrane surface these monomers self assemble, in an ATP-independent process, into the functional heptamer geometry. The oligomerization that completes the formation of the heptamer provides the energy to punch through the membrane to form the highly stable alpha-Hemolysin channel. From crystallographic results [1], we know that the alpha-hemolysin water filled channel ranges in diameter from 2.6 nm at the *cis*-side opening to 1.5 nm at the limiting aperture. The length of the channel along its line of axial symmetry is approximately 10 nm. The channel widens in the middle creating a chalice shaped cross section along its axis. This channel widening provides a cavity for a captured molecule to wiggle about. Many different molecules have been examined on the nanopore detector platform, including biopolymers like ssDNA, dsDNA, ethylene glycol, and a variety of sugars and proteins (see Background for more details).

Previous nanopore detector measurements involving hairpin DNA molecules with varying base stem lengths have shown a relationship between the number of base pairs and the occurrence of a bi-level dominated current signal or "toggle signal" [2]. These experiments also serve to directly confirm the channel geometry described above, where the DNA hairpins can be viewed as "depth gauges" of varying length. A model for the mechanism of the toggle signal, that is observed for 9 base pair DNA hairpins, is proposed as an interaction between the terminus of the DNA hairpin stem and the limiting aperture's border amino acids (see [3]). Upon introduction of antibodies to the same system, similar blockage signals have been observed suggesting a similar mechanism is responsible for the antibody toggle signal.

### Nanopore blockade detector

**Figure 1:**
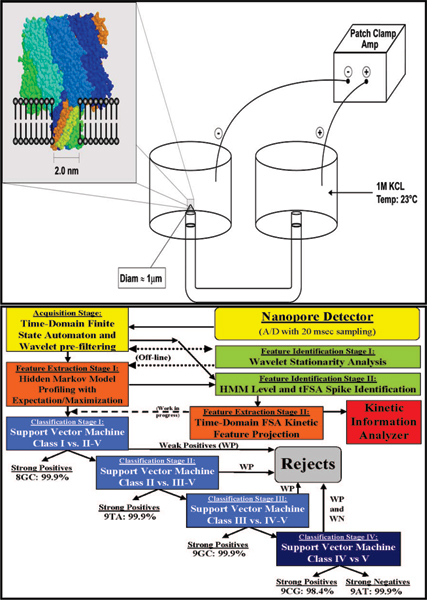
**Single-nanopore based channel current analysis and detection**. A nanometer-scale channel can be used to associate ionic current measurements with single-molecule channel blockades (Fig. 1, Top). The **α**-hemolysin channel self-assembles, leading to an inexpensive and reproducible nanopore detector. The **α**-hemolysin based nanopore detector is well-suited to observation of biomolecular kinetics that can modulate ionic flow through its limiting aperture. The signal processing architecture is shown in the Bottom Panel. See [4] for details on the cheminformatics architecture.

There is an important distinction in how a nanopore detector can function, described here as direct vs. indirect measurement of molecular event statistics. It is possible for a nanopore-based detector to *directly *infer molecular event statistics from the blockade properties of individual molecules [3,4]. There are two distinct approaches, one based on inducing nanopore translocation events, and their channel-current modulations, the other based on vestibule-captured, but non-translocating, events, and their channel current modulations. For non-translocating molecules, we have a much more informative setting, based on the kinetic information that is embedded in the blockade measurements, where the adsorption-desorption history of the molecule to the surrounding channel, and the configurational changes in the molecule itself, can significantly and directly imprint on the ionic flow through the channel [2-7], see Fig. 1, Top Panel.

The original and prevailing method of characterizing DNA oligonulceotides is based on analyzing the depth and duration of the static channel blockade created by ssDNA freely passing, also referred to as "translocating," through the channel [8]. The method employed in this study and similar studies is quite different in that the shape of our specially designed dsDNA molecules makes them unable to fully translocate, and thus, the blockade signal produced corresponds to that of a (partially) trapped, non-translocating molecule [2-7,9,10]. The direct approaches offer prospects for DNA sequencing (via translocation observations [8,11-19]) and single nucleotide polymorphism (SNP) analysis (via non-translocation observations [2-7]). In one direct study of molecular event statistics [11], the binding of an individual DNA oligonucleotide, covalently tethered within the lumen of the alpha-hemolysin pore to free-floating ssDNA, caused changes in the ionic current flowing through a nanopore that allowed discrimination between individual DNA strands up to 30 nucleotides in length. This is a very brief and limited synopsis of the Nanopore Detector background relevant to this paper. For other references on Nanopore Detectors use is made of a Nanopore Detector review presented in [12]: early work involving alpha-Hemolysin Nanopore Detectors can be found in [2-7,11,13-19]; rapidly growing research endeavors on Nanopore Detectors based on solid-state, and other synthetic, platforms can be found in [20-30].

**Figure 2:**
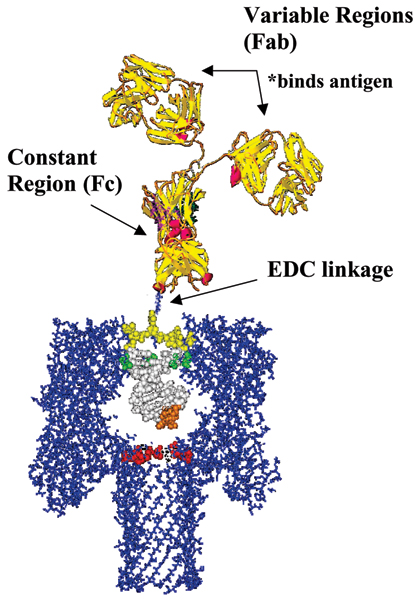
**Linked Antibody Experiment [10]**. Approximately shown to scale on the left is the DNA-antibody complex and its intended captured orientation in the alpha-hemolysin channel. An internal amino thymine modification or phosphate backbone modification with primary amine on a six carbon spacer arm is crosslinked using 1-Ethyl-3-(3-dimethylaminopropyl) carbodiimide hydrochloride (EDC) to the peptide carboxyl terminus of the antibody heavy chain. This crosslinkage results in a covalent bond between the primary amine and the carboxyl terminal.

The nanopore-based detector works *indirectly *if it uses a reporter molecule that binds to certain molecules, with distinctively new blockade statistics for the bound-molecule complex [9,10]. Figure 2 shows such an indirect detection platform, with a carefully designed DNA hairpin to blockade, and provide a highly sensitive "toggle" signal. The DNA hairpin, in turn, is linked to an antibody, which provides the specific binding function – in this case to the antibody's target antigen. Indirect statistical measurement sees durations of one blockade statistic profile, then another, the phase of which corresponds to the lifetime of a state (bound or unbound, for example). Contrast this with the direct statistical measurement of state information – the fixed blockade levels correspond directly to the states of interest, something directly modulating the channel limiting aperture environment, for example. Such indirect observation of binding, or event transduction detection, is explored here in the case of antibody-antigen binding studies, where the antibody serves as reporter molecule.

A recent example of indirect statistical observation, the biologically relevant interactions between ssDNA and a protein, the bacterial enzyme Exonuclease I, were examined [31]. If the free-floating ssDNA bound the enzyme in solution, it then formed a complex too large to pass through the channel. The ssDNA remained in the channel until it finally detached from the enzyme. The time it takes for the bonds between the DNA and the enzyme to break informs about the nature of their kinetic interaction. Contrast the [31] indirect kinetic study with the direct kinetic study done by [11], where kinetic features are directly measured in the sense that the molecular event presented there directly modulates the channel environment flow, and with [31] it does so by indirectly releasing the molecule and allowing it to translocate.

### Bifunctional immunoglobulins

The immunoglobulin molecule IgG is often described as a bifunctional molecule: one region for binding to target antigen, the other region for mediating effector function. Effector functions include binding of the antibody to host tissues, to various cells of the immune system, to some phagocytic cells, and to the first component (C1q) of the classical complement system. Activation of the immune system in response to a specific antigen is an amazing example of how a series of protein phosphorylation and dephosphorylation reactions convert a cell surface event to changes in DNA transcription and cell replication.

The structure of the IgG antibody forms three globular regions that are attached to each other in the middle of its grouping (see image of IgG embedded in Fig. 2). The overall shape of the structure forms a Y configuration. At the base of this structure is the Constant (Fc) region where the effector functions take place and at the tips of the two arms, both referred to as the variable region (Fab), are the antigen binding sites. These variable regions are tethered to the trunk of the Y shaped molecule by a flexible hinge which allows for a high degree of arm movement. The relative size of the antibody is about three times the size of the alpha-hemolysin channel. Its length from base (Fc) to arm tip (Fab) is 25 nm and the width of each globular arm ranges from 6–10 nm [34-37].

The forces binding antigen to antibody are an important and difficult area of study. Hydrophobic bonds, in particular, are very difficult to characterize by existing crystallographic and other means, and often contribute half of the overall binding strength of the antigen-antibody bond. Hydrophobic groups of the biomolecules exclude water while forming lock and key complementary shapes. The importance of the hydrophobic bonds in protein-protein interactions, and of critically placed waters of hydration, and the complex conformational negotiation whereby they are established, may be accessible to direct study using nanopore detection methods in future developments of this technology. In the preliminary work that follows, however, we focus on the recognition of a binding event, not the nature of that binding event (future work to decipher the binding mechanism will require far more extensive data gathering and analysis and will not be discussed further).

## Results

### Ab-antigen binding and Ab-pore interaction

**Figure 3:**
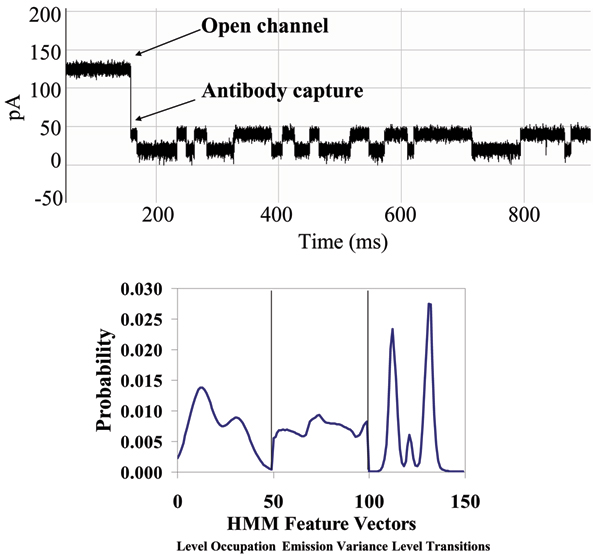
**Antibody toggle signal and HMM signal profile. **On the left is capture event of an antibody molecule which has yielded a toggle signal. The first portion of the signal is at the 120 pA current reading, standard for an open alpha-hemolysin channel. A sudden drop of the ionic current indicates a physical blockade of the channel has occurred reducing its current flow. On the right: An HMM is used to remove noise from the acquired signals, and to extract features from them. The HMM is implemented with fifty states. Each blockade signature is de-noised by 5 rounds of Expectation-Maximization (EM) training on the parameters of the HMM. After the EM iterations, 150 parameters are extracted from the HMM. The 150 feature vectors obtained from the 50-state HMM-EM/Viterbi implementation are: the 50 dwell percentages in the different blockade levels from the Viterbi trace-back states, the 50 variances of the emission probability distributions associated with the different states, and the 50 merged transition probabilities from the primary and secondary blockade occupation levels (meant to work well with two-state dominant modulatory blockade signals)**.**

**Figure 4:**
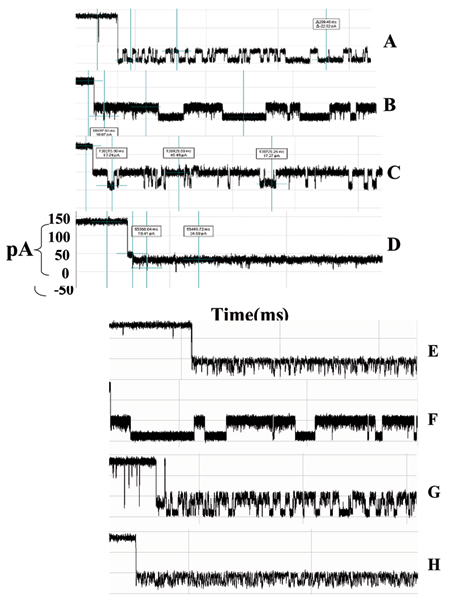
****Antibody Signal Classes and Ab-Antigen Signal Classes**. **A-D: various IgG region captures and their associated toggle signals (1 second traces). E-F: various IgG+Antigen region captures and their associated toggle signals (1 second traces). Each blockade signal was identified visually and represents a commonly observed signal class. Note the changes in dwell times for the upper and lower current levels in each signal class. We find a higher current level bias in the level occupancy as a result of binding with the antigen molecule**.**

**Figure 5:**
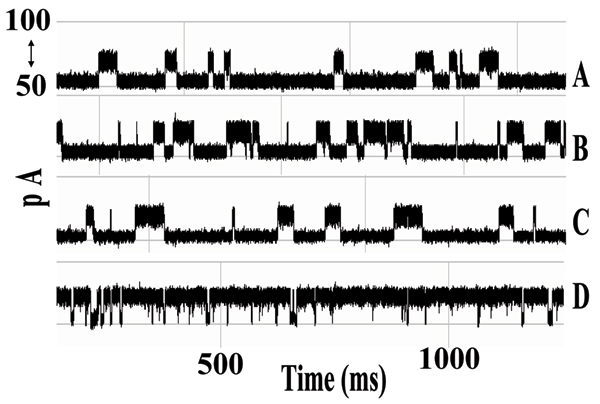
****Antibody-Antigen Binding Event**. **An antibody molecule has been captured by the Alpha-hemolysin channel producing a clear and continuous toggle signal. Antigen is introduced to the 70 ul *cis *well of the nanopore chamber in frame (A). Subsequent data files containing toggle signals of three minute intervals are recorded and displayed as B, C, and D (only the first second of each 3 minute phase is shown). Changes to the toggle signal are detected in frames C and D indicating the binding event between the antibody and antigen has taken place.

**Figure 6:**
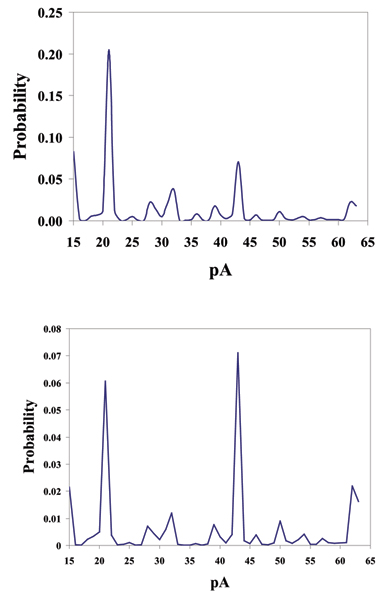
****Multivalent antigen binding**. **Top Panel**: **First Antibody Antigen Binding – 1^st ^50 feature components extracted from the HMM in Fig 2. **Bottom Panel**: Shifts in the values of these 1^st ^50 HMM feature components indicate a possible second Antibody Antigen Binding (same molecular capture). The first 50 components of the 150 feature vectors obtained from the 50-state HMM-EM/Viterbi implementation are the dwell percentages in the different blockade levels from the Viterbi trace-back states (approximately the Histogram in that range).

In the presence of IgG we observed partial blockage of the ionic current through the pore. The observed toggle signal, familiar from the DNA hairpin studies, is the focus of our study. Repeated experiments showed capture events offering a variety of signal types. These current blockade events had the general characteristics of those produced by hairpin DNA molecules. Antibody capture events were easily ejected with reversal of ionic current from 120 mV to -120 mV. Often these captures self ejected after a brief period of time ranging from a few milliseconds to a few minutes, although we have observed capture toggle signals that endured for as long as 30 minutes or more. Albumin, Streptavidin, Biotin, and murine IgG1 antigen have been introduced into the nanopore chambered resulting in little to no interactions with the channel and none displayed lengthy or toggle-like current blockades. We have tested IgG subclass 1 monoclonal antibodies for biotin, HIV, and anti-GFP, and all have produced similar signals. Due to the strong similarity between toggle signals generated with a DNA hairpin and signals generated by an antibody molecule, we use the same terminology with the assumption that a similar process of current blockade is taking place (see Fig. 3). We have accumulated hundreds of capture events displaying the variety of capture signals, and representative signals are shown in Fig. 4 (The representative signals are chosen manually at this time since it is easily managed in this instance. An objective SVM-based identification of "representative" signals is possible, however, and has been used in the past [4].). Additionally we have recorded changes in these signals after introduction of the binding element (antigen), indicating sensitivity to changes in states due to the binding of these proteins (see Fig. 5). In Fig. 6, Top Panel, we have the HMM profile for the first binding event (the channel current cheminformatics methods are briefly described next). A change is then seen during that same capture event to the HMM profile shown in the Bottom Panel. This is thought to be evidence of a second binding event on the antibody's other arm, but numerous repetitions of such events will be needed to confirm this.

### Channel current cheminformatics

The signal processing architecture (Fig. 1, Bottom Panel) is designed to rapidly extract useful information from noisy blockade signals using feature extraction protocols, wavelet analysis, Hidden Markov Models (HMMs) and Support Vector Machines (SVMs). For blockade signal acquisition and simple, time-domain, feature-extraction, a Finite State Automaton (FSA) approach is used [32] that is based on tuning a variety of threshold parameters. A generic HMM can be used to characterize current blockades by identifying a sequence of sub-blockades as a sequence of state emissions [2-7]. The parameters of the generic HMM can then be estimated using a method called Expectation/Maximization (or 'EM") [33], to effect de-noising. The HMM method with EM, denoted HMM/EM, is used in what follows (further Background on these methods can be found in [2-7]). Classification of feature vectors obtained by the HMM for each individual blockade event is then done using SVMs, an approach which automatically provides a confidence measure on each classification.

### Indirect detection experiments

**Figure 7:**
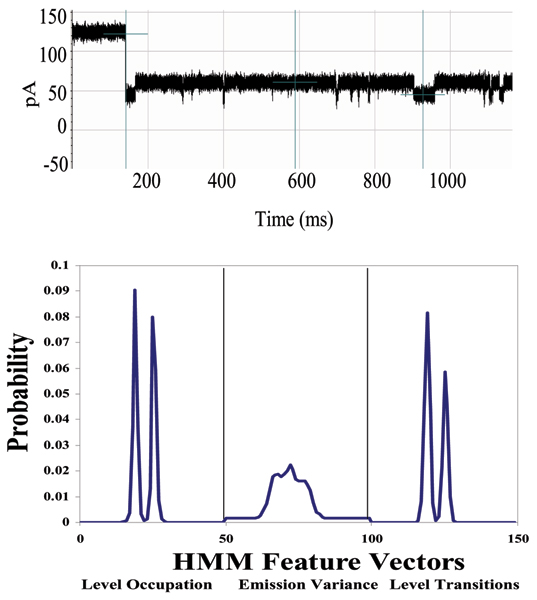
****Top**: **Biotinylated DNA Hairpin Captured in the Nanopore Detector. **Bottom**: Biotinylated DNA Hairpin HHM-based CCC Analysis. HMM Signal Profile: The 150 feature vectors obtained from the 50-state HMM-EM/Viterbi implementation in [4-7] are: the 50 dwell percentage in the different blockade levels (from the Viterbi trace-back states), the 50 variances of the emission probability distributions associated with the different states, and the 50 merged transition probabilities from the primary and secondary blockade occupation levels (fits to two-state dominant modulatory blockade signals). The first 50 features, corresponding to the dwell times are, effectively, a de-noised histogram of the blockade samples seen in the "active" window between 20% and 70% of the open channel; current**.**

**Figure 8:**
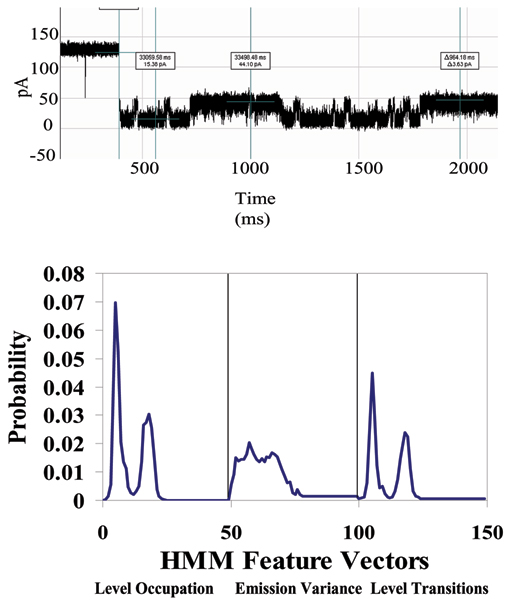
**Top**: signal and profile after introduction of an excess of streptavidin – a binding event between streptavidin and the biotinylated hairpin is hypothesized to be the cause of the significant shift in the HMM feature vectors, shown in the **Bottom Panel**. The signal is only observed after introduction of streptavidin, leading us to hypothesize its possible direct association with a binding event. The HMM Signal Profile: The 150 feature vectors obtained from the 50-state HMM-EM/Viterbi implementation in [4-7] are: the 50 dwell percentage in the different blockade levels (from the Viterbi trace-back states), the 50 variances of the emission probability distributions associated with the different states, and the 50 merged transition probabilities from the primary and secondary blockade occupation levels (fits to two-state dominant modulatory blockade signals).

In order to emulate nature's bifunctional molecule (the antibody), we have biotinylated a hairpin DNA molecule, targeted by Streptavidin, to create the first in a series of linked DNA complexes designed as nanopore reporters for protein-protein interactions. The DNA has a modified thymine inserted in the top of its hairpin loop. A six carbon spacer arm extends perpendicularly to the axis of the DNA, allowing for unhindered biotin interaction. This thymine addition to the four thymine loop of the hairpin structure slightly modifies the toggle signal from that of the four thymine loop, but unlike the unmodified five thymine loop hairpin no deep insertions are observed. Hairpin structures containing more than four thymine in their loops often produce deeper current blockages. It is assumed that the added flexibility of a five or more thymine loop, being less sterically hindered allows loop folding to occur permitting deeper channel entry. The modified thymine loop hair pin produces a repeatable capture signal. The streptavidin-biotin test case (the strongest biochemical interaction known) shows clear binding evidence (see Figs. 7 &8), and also clarifies what we might expect for observations of binding in this setting.

**Figure 9:**
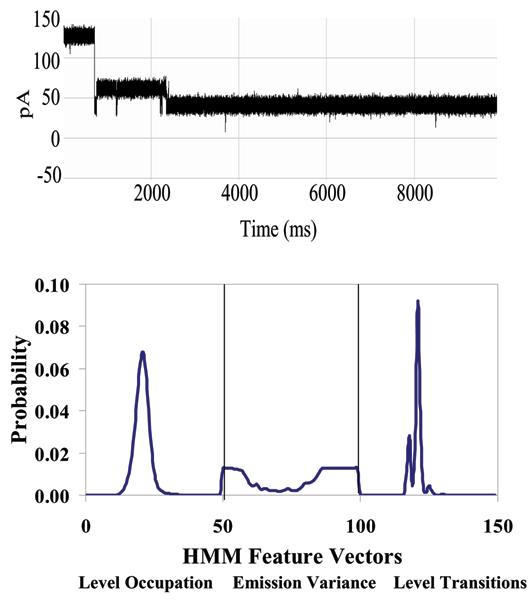
**Linked Antibody Experiment [10]**. The unique carrier signal is displayed in the Top Panel for the DNA-Antibody blockade. In the Bottom Panel, the HMM feature profile is shown.

**Figure 10:**
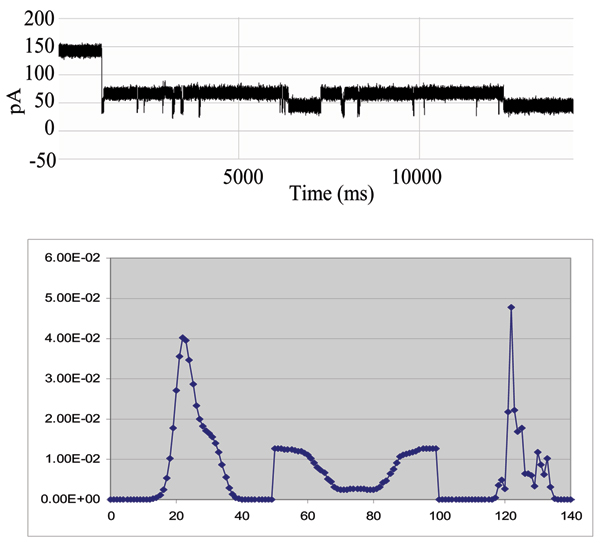
**Linked Antibody Experiment [10]**. After introduction of target antigen (biotin), a new signal class is obtained – see the signal example in the Top Panel and the HMM signal profile in the Bottom.

**Figure 11:**
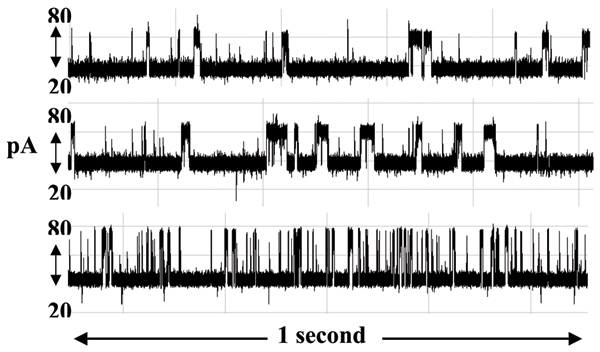
**Antibody blockades in the presence of antigen excess and chaotropic agents**. An antibody (anti-biotin) molecule is introduced to the nanopore device to produce the characteristic signal shown in panel 1. In present of the antigen (biotin) there is a strong interaction: even 100 fold excess of biotin does not change signal considerably (panel 2). The signal changes greatly in presence of urea (panel 3), in relatively small concentrations insufficient to result in protein denaturing. Signal durations are shown for one second.

A DNA hairpin with EDC linkage to an antibody is studied next (see Figs. 2, 9, and 10), with Antibody-Antigen binding experiments via the linker setup examined in [10]. When the DNA portion of this linked complex inserts itself into the alpha hemolysin channel it creates a definable toggle signal that serves as reliable "carrier signal" for monitoring any changes of molecular state (such as binding). In our first study of DNA-hairpin linked antibody complexes [10], we used an anti-biotin-antibody (Stressgen) as our binding element linked to our DNA hairpin. Displayed in Fig. 11 is the toggle signal after addition of excess biotin. The *small*-antigen biosensing results (Figs. 2, 9, 10, 11) complements those in Fig's. 3, 4, 5, 6 for *large*-antigen biosensing (with the antibody binding to the large polypeptide antigen described in the Methods). The large antigen study done here also differs in that it involves direct use of the antibody as a bifunctional reporter molecule. This leads to complications with capture, and uniqueness in the orientation of that capture, but may offer a more sensitive detection since there is not a linkage separating the bound/unbound complex from the channel flow environment.

### Ab-antigen binding and Ab-pore interaction in presence of urea and MgCl_2_

The clarity of the current blockade signal was examined by varying the composition of working buffer. In one series of experiments, mentioned above, we used free antibody molecule interacting with the nanopore detector, where the antibody (anti-biotin) molecule is introduced to our nanopore device to produce the characteristic two-state telegraph signal (Fig. 11). The blockade signal for the antigen is practically unaltered by excess antigen: even 100 fold excess of biotin does not change the blockade signal considerably (Fig. 11, panel 2). The signal changes greatly in presence of urea, however, in a relatively small concentration. Here the duration of any event to occupy upper state level becomes shorter and the total probability value of upper level decreases with urea concentration rise. In the other series of experiments the antibody was conjugated to the 9GC DNA hairpin. Primary amine crosslinked using 1-Ethyl-3-(3-dimethylaminopropyl) carbodiimide hydrochloride (EDC) to the peptide carboxyl terminus of the antibody heavy chain. This crosslinkage results in a covalent bond between the primary amine and the carboxyl. With this arrangement, two transient states were observed in the current signal resulting from the IgG-DNA molecule blockades (see figure in Additional File 1). Additional File 1 shows the Current blockade signal change induced by presence MgCl_2 _(0.4 M). Antigen binding to Ab-DNA hairpin appears to result in a more complex signal (lower panel). With MgCl2 concentration increase, there is an increased occurrence of the upper-level blockade state, at the same time the current signal becomes nosier and the open channel current increases to 130 pA. The lower panel corresponds to 0.4 M concentration of MgCl_2_. The total time scale is 15 seconds.

With progressive MgCl_2 _concentration, there exist a tendency of higher probability for the upper state, at the same time the current signal becomes noisier and the open channel current rises up to 130 pA. The effect of increase of KCL on a DNA hairpin blockade signal is shown in the figures in Additional Files 1 and 3. Additional File 2 shows the current blockade signal change with KCl increase. A nine base-pair DNA hairpin with a distinctive upper level toggle is shown in the top panel. The middle and bottom panels show the blockade patterns at 1.9 and 2.5 M KCl, correspondingly. As the concentration of background electrolyte increases, the signal keeps its highly structured profile, although becoming noisy. Additional File 3 shows the "Upper level toggler" DNA hairpin profiles at different KCl concentrations. The 150-component profiles of the signals for a nine base-pair DNA hairpin, known to exhibit a "fine-structure" in its upper-level, at 1.0, 1.9 and 2.5 M KCl (corresponding to the signal shown in the previous image). Each blockade signature is de-noised by 5 rounds of Expectation-Maximization (EM) training on the parameters of the HMM. After the EM iterations, 150 parameters are extracted from the HMM. The 150 feature vectors obtained from the 50-state HMM-EM/Viterbi implementation in are: the 50 dwell percentage in the different blockade levels (from the Viterbi trace-back states), the 50 variances of the emission probability distributions associated with the different states, and the 50 merged transition probabilities from the primary and secondary blockade occupation levels (fits to two-state dominant modulatory blockade signals).

## Discussion

A discrete level change after initial current blockade is a distinctive feature of channel blockade detection at this scale (as previous work with hairpin DNA has shown [2-7]) and supports the assumption of protein-loop or terminus insertion into the channel as a possible mechanism for this process. Assuming that a very small portion of the globular antibody has inserted itself into the channel, this type of signal could be produced by a protein branch dangling over the limiting aperture. The DNA hairpin molecules studied in [2-7] produce such a signal as its nine base pair stem dangles over the limiting aperture typically creating a two state dominant current blockade. The fixed blockade levels are thought to represent short lived ionic bonds forming between the residues of the alpha-hemolysin channel and the terminal base pair of the hairpin stem. The Hidden Markov Model (HMM) signal profiles in the figures, have as their first 50 components, a histogram-like profile of the main blockade region that usually resides between 40% and 60% of the baseline current.

The IgG antibody may vary in net charge and is nowhere near as negatively charged as the DNA hairpin molecules examined. Differences in channel interaction are often attributed to its net charge and its electrophoretic mobility [2,16]. To improve the antibody's affinity for the channel and to aid in signal classification, a complex of antibody and DNA hairpin is linked together. The result is the increase in channel affinity and a significant reduction in capture class configurations (see Figs. 2, 9, 10 and [10] for further details), while, evidently, still retaining binding detection sensitivity.

For the antigen-binding studies, different versions of copolymer (Y, E)-A – K are prepared which vary in molecular weight and valency (the one-letter codes are for respective the amino acids). Previous studies have demonstrated that the epitope to which the antibodies bind may be represented by the synthetic peptide EYYEYEEY [34-37]. Thus we have the following antigens: 1. The synthetic polypeptide (Y, E)-A – K, which is highly multivalent and with different preparations has a molecular of either 50,000, 125,000, or 300,000 daltons. 2. EYYEYEEY which is monovalent and has a molecular weight of 1183, and 3. Ovalbumin, molecular weight 43,000 which has been conjugated to EYYEYEEY at ratios of 1, 3, and 10 peptides per molecule of OVA. These different antibody preparations allow study of the effect of antigenic mass and valency of binding upon the observations.

For the experiments on antibody (anti-biotin) interaction with alpha-hemolysin channel, we assume as a possible interpretation that the hypervariable loops of the antibody can be captured by the nanopore. Since the blockade signal for the antigen is practically unaltered by excess antigen, even the 100 fold excess of biotin does not change the blockade signal considerably (see Results), biotin is not thought to interact greatly with the (blocked) channel. The strong signal changes observed in presence of urea and MgCl_2_, in relatively small concentrations normally insufficient to result in protein denaturing, on the other hand, allows us to hypothesize significant channel-Ab binding interference by urea.

## Conclusion

Experimental efforts have found that different forms of channel blockade can be associated with the same antibody, presumably associated with different orientations of "capture" of an antibody in the detector's nanometer-scale channel (i.e., the antibody presents numerous epitopes for nanopore capture). To get around the non-specific capture orientation limitation, DNA-hairpin anchored antibodies have also been an area of study [10], where the DNA linkage is to the carboxy-terminus at the base of the antibody. On-binding is easily observed in all of the above experiments, as shown in the Results. So the difficulty is the observation of Off-binding in the timescale of the experiment. For this reason weaker binding-pairs are sought, or are being induced by introduction of divalent ions (MgCl_2 _is added to the buffer, for example) and chaotropic agents in general.

In addition to a murine IgG1, a human IgG1, and a human IgG4 (see Methods for details), we plan on testing the following antibodies: murine and human monoclonal antibodies representing all of the IgG isotypes, murine IgG1 antibodies having different pIs (determined experimentally), and polyclonal protein A purified antibodies from human, rabbit, goat and mouse. These and other antibodies are going to be used to determine whether the ability of antibodies to be captured is a general phenomenon, and whether the blockade patterns obtained with antibodies are dependent on pI, isotype, species origin, or other predictable characteristics of the antibody or of the method of preparation.

Insofar as an analysis of the binding affinity between two complex proteins is concerned (like the antibody and its target antigen), this study also provides a test case for nanopore-based protein-protein interaction studies. It might prove possible to rank the different antibody binding strengths to target antigen, for example, according to the observed lifetimes of their bound states. Efforts are underway to observe full binding histories to get very precise measurements of K_on _*and *K_off_. Nanopore-based approaches may eventually provide a direct analysis of the complex conformational "negotiations" that occur upon binding between proteins.

## Methods

### Nanopore experiments

Each experiment is conducted using one α-hemolysin channel inserted into a diphytanoyl-phosphatidylcholine/hexadecane bilayer across a 25-micron-diameter horizontal Teflon aperture, as described previously [2-4] (Fig. 1). Two seventy microliter chambers on either side of the bilayer contain 1.0 M KCl buffered at pH 8.0 (10 mM HEPES/KOH), except in the case of buffer experiments where the salt concentration, pH, or other aspects of composition may be varied (introduction of glucose, urea, etc.). Voltage of 120 mV is applied across the bilayer between Ag-AgCl electrodes. DNA control probes are added to the *cis *chamber at 10 or 20 μM final concentration. All experiments are maintained at room temperature (23 ± 0.1°C), using a Peltier device.

### Control probe design

Since the five DNA hairpins studied in the prototype experiment have been carefully characterized [4], they are used in the antibody (and other) experiments as highly sensitive controls. (The probes are used, periodically, to test the channel for proper operation, or if a channel is suspected to be performing abnormally, they are used to test right away. If recognized blockade signals don't result upon introduction of control it is assumed that the channel is not operating under desirable conditions.) The nine base-pair hairpin molecules examined in the prototype experiment share an eight base-pair hairpin core sequence, with addition of one of the four permutations of Watson-Crick base-pairs that may exist at the blunt end terminus, i.e., 5'-G•C-3', 5'-C•G-3', 5'-T•A-3', and 5'-A•T-3' denoted 9GC, 9CG, 9TA, and 9AT, respectively. The full sequence for the 9CG hairpin is 5' CTTCGAACGTTTTCGTTCGAAG 3', where the base-pairing region is underlined. The eight base-pair DNA hairpin is identical to the core nine base-pair subsequence, except the terminal base-pair is 5'-G•C-3'. The prediction that each hairpin would adopt one base-paired structure was tested and confirmed using the DNA mfold server .

### Antibody/antigen design, synthesis, and purification

For most of the experiments, a panel of native and genetically engineered antibodies to a well defined synthetic polypeptide antigen are used, (Y, E)-A – K, [34-37]. The antigen-binding characteristics, ability to form immune complexes, and effector functions of these antibodies have been carefully studied. Three different antibodies from this set are utilized in the experiments in this experimental effort. All have identical variable domains of murine origin, but one is a murine IgG1, one a human IgG1, and the other a human IgG4. Only the murine IgG1 data is shown in this paper.

All monoclonal antibodies are grown in tissue culture because ascites preparations are inflammatory exudates subjecting the antibodies to the potential of proteolytic digestion, attachment of complement components and so forth. Cells are either grown in medium containing fetal calf serum adsorbed on protein G to remove remaining Ig, or in serum free hybridoma medium. To test the effect of preparation method, murine IgG1 antibody is either purified by ammonium sulfate precipitation, antigen-affinity purification or protein G chromatography. All other antibodies are routinely purified on protein G and eluted with 0.5 M glycine-HCl pH 2.5, immediately neutralized, and dialyzed into phosphate buffered saline (PBS). Once antibodies are purified, they are run on SDS-PAGE to confirm purity and run on IEF prepoured gels (Biorad) to determine PI. Antigen binding is confirmed by the immunoassay technique of ELISA (enzyme-linked immunosorbent assay, a biochemical technique to identify the presence of antibody or antigen) in PBS and in 1 M KCl (so long as that buffer is used).

### Mouse anti-Biotin monoclonal antibody

Anti-Biotin Antibody monoclonal IgG1 from Stressgen was used at the concentration of 1.0 mg/mL. Horseradish peroxidase (HRP) was conjugated with affinity purified mouse immunoglobulin in phosphate buffered saline (PBS) at pH 7.2 with 0.1 mM PMSF and 50% glycerol. The Immunogen was un-bound Biotin.

## Competing interests

The authors declare that they have no competing interests.

## Authors' contributions

The paper was written by SWH. The core feature extraction and pattern recognition software was developed by SWH. The nanopore experiments involving antibody and antibody-antigen were performed by EM. The nanopore experiments exploring buffer changes were performed by AS.

## Supplementary Material

Additional file 1 Antigen  binding to Ab-DNA hairpin  appears to result in a more complex signal (lower panel).  With  MgCl2 concentration increase, there is an increased occurrence of the upper-level blockade state, at the same time the current signal becomes nosier and the open channel current increases to 130 pA.  The lower panel corresponds to 0.4M concentration of MgCl2.  The total time scale is 15 seconds. Click here for file

Additional file 2 A nine base-pair DNA hairpin with a distinctive upper level toggle is shown in the top panel (at 1M KCl). The middle and bottom panels show the blockade patterns at 1.9 and 2.5 M KCl, correspondingly Click here for file

Additional file 3 The 150-component profiles of the signals for a nine base-pair DNA hairpin, known to exhibit a “fine-structure” in its upper-level, at 1.0, 1.9 and 2.5 M KCl (corresponding to the signal shown in the previous image). Click here for file
