# Supplementary figures and images for: Nanopore-based kinetics analysis of individual antibody-channel and antibody-antigen interactions
Source: BMC Bioinformatics. 2007 Nov 1;8(Suppl 7):S20. doi: 10.1186/1471-2105-8-S7-S20 (PMC2099489; doi:10.1186/1471-2105-8-S7-S20)

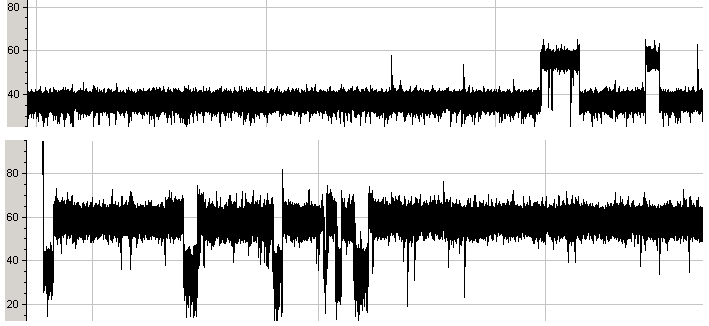


**15 seconds**

**pA**

**80**

**60**

**40**

**80**

**60**

**40**

**20**

Supplement: Additional file 1 — Antigen binding to Ab-DNA hairpin appears to result in a more complex signal (lower panel). With MgCl2 concentration increase, there is an increased occurrence of the upper-level blockade state, at the same time the current signal becomes nosier and the open channel current increases to 130 pA. The lower panel corresponds to 0.4M concentration of MgCl2. The total time scale is 15 seconds. [file 1471-2105-8-S7-S20-S1.doc]

**2 seconds**


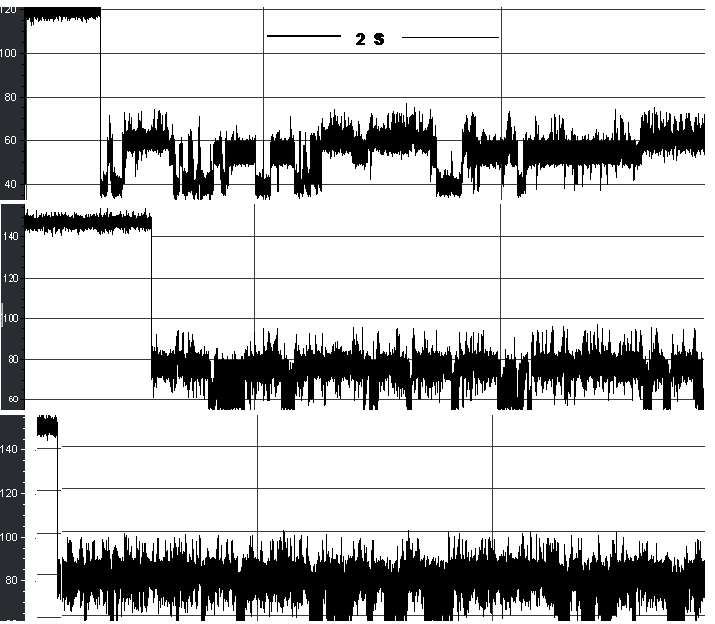


**120**

**155**

**80**

**147**

**60**

**80**

**pA**

**(1)**

**(3)**

**(2)**

**Time**

Supplement: Additional file 2 — A nine base-pair DNA hairpin with a distinctive upper level toggle is shown in the top panel (at 1M KCl). The middle and bottom panels show the blockade patterns at 1.9 and 2.5 M KCl, correspondingly [file 1471-2105-8-S7-S20-S2.doc]
