# Supplementary material for: Nanopore-based kinetics analysis of individual antibody-channel and antibody-antigen interactions
Source: BMC Bioinformatics. 2007 Nov 1;8(Suppl 7):S20. doi: 10.1186/1471-2105-8-S7-S20 (PMC2099489; doi:10.1186/1471-2105-8-S7-S20)
Supplement: Additional file 3 — The 150-component profiles of the signals for a nine base-pair DNA hairpin, known to exhibit a “fine-structure” in its upper-level, at 1.0, 1.9 and 2.5 M KCl (corresponding to the signal shown in the previous image). [file 1471-2105-8-S7-S20-S3.doc]

**50**

**100**

**0**

**150**

**Level Occupations**

**Emission Variances**

**Level Transitions**

**2.50M**

**1.00M**

**1.85M**
